# Single-cell and spatial transcriptomics reveal a stress-induced EMT-like epithelial subset driving immune activation in silica-injured lung

**DOI:** 10.3389/fimmu.2025.1609616

**Published:** 2025-06-06

**Authors:** Tao Wang, Jianfeng Hao, Bing Li, Ahjol Hyraht, Jialing Wang, Henglei Xia, Qingbin Wu, Wei Gao, Congxia Chen, Chuanqing Yu, Xiuqun Gong, Ting Li, Mei Zhang, Yinghai Xie, Xinrong Tao

**Affiliations:** ^1^ The First Hospital of Anhui University of Science and Technology (Huainan First People’s Hospital), Huainan, Anhui, China; ^2^ School of Public Health, Anhui University of Science and Technology, Hefei, Anhui, China; ^3^ School of Medicine, Anhui University of Science and Technology, Huainan, Anhui, China; ^4^ State Key Laboratory of Primate Biomedical Research, Institute of Primate Translational Medicine, Kunming University of Science and Technology, Kunming, China; ^5^ Kunshan Integrated Traditional Chinese and Western Medicine Hospital, Kunshan, Jiangsu, China

**Keywords:** silicosis, alveolar epithelial cells, single-cell RNA sequencing, spatial transcriptomics, epithelial-immune crosstalk

## Abstract

The mechanism that lung epithelial cells regulate immune responses during chronic injury still remains unclear. Here, we combined single-cell RNA sequencing with spatial transcriptomics to track epithelial dynamics in silica (SiO_2_)-exposed mouse lungs. By day 56, SiO_2_ induced significant epithelial proliferation, followed with a distinct C0 subset emerging as the dominant population. C0 cells co-expressed surfactant genes (*Sftpc*, *Scgb3a2*), mesenchymal markers (*Vim*, *Mmp12*), and pro-inflammatory cytokines (*Ccl6*, *S100a8/a9*), reflecting a hybrid phenotype. Spatial and cell-cell interaction analyses showed C0 cells engaging macrophages and neutrophils through SPP1-CD44, APP-CD74, and GRN-MARCO signaling, driving immune recruitment and activation. Pseudotime and CytoTRACE analyses indicated that C0 cells represent a late-stage, low-stemness state with epithelial-mesenchymal transition (EMT)-like features. Taken together, these findings reveal a novel, stress-induced epithelial subset that amplifies immune crosstalk and tissue remodeling, offering new perspectives on silica-induced lung injury.

## 1 Introduction

Lung injury and inflammation resulting from environmental or occupational exposure to silica (SiO_2_) remain significant challenges in clinical practice and scientific research, particularly in industrial and mining settings. Silica dust, a pervasive environmental pollutant, is a well-known cause of lung diseases such as pneumoconiosis (1), which often leads to pulmonary fibrosis, a disease characterized by excess collagen buildup and permanent lung scarring. Globally, tens of millions of workers remain exposed to silica dust across various industries, including mining, construction, and engineered stone manufacturing. For instance, China reported approximately 9,000 new cases annually due to extensive silica exposure in the mining and construction industries, while India and Brazil have over 10 million and 6 million workers respectively who are regularly exposed to silica dust (2). Moreover, occupational silicosis is responsible for more than 12,900 annual deaths and approximately 0.65 million disability-adjusted life years worldwide (2).

At the cellular and molecular levels, SiO_2_ induces apoptosis in lung epithelial cells and stimulates epithelial cells to release exosomes that promote collagen deposition and activate lung fibroblasts (3, 4). Another effect of SiO_2_ is that it induces a proinflammatory response in pulmonary immune cells. For instance, exposure to SiO_2_ suppresses pulmonary T-cell responses and induces a shift in macrophage polarization from a resting (M0) state to a proinflammatory (M1) phenotype (5–7). Macrophages, through the phagocytosis of SiO_2_ particles, trigger a cascade of proinflammatory and profibrotic responses. More recently, lung epithelial cells have gained attention for their role in innate and adaptive immunity, mediated by the secretion of cytokines (8). Despite these insights, the precise mechanisms underlying epithelial cell behavior and their interplay with immune cells following SiO_2_ exposure remain poorly understood, underscoring the need for further research.

Central to unraveling these mechanisms is the role of epithelial cells in the lung’s response to SiO_2_-induced stress. The alveolar epithelium, a critical structural and functional barrier, comprises two main cell types: type I alveolar cells (AT1), which mediate gas exchange across most of the alveolar surface, and type II alveolar epithelial cells (AT2), which produce surfactant to prevent alveolar collapse and support repair by proliferating and differentiating into AT1 cells following injury (9, 10). Repeated epithelial injury from chronic SiO_2_ exposure disrupts these repair processes, potentially resulting in aberrant cellular responses such as epithelial-to-mesenchymal transition (EMT), which further promotes fibrosis. Notably, epithelial cells exposed to stressors like SiO_2_ express specific gene programs that profoundly influence their interactions with immune cells, balancing inflammatory and repair processes. For example, in the lung injury model, exogenous SCGB3A2 was shown to accelerate tissue repair and mitigate fibrosis by modulating TGF-β signaling (11, 12). Similarly, other evidence indicates that SPP1, highly expressed in the lung tissue of pulmonary fibrosis patients, promotes M2 macrophage polarization and induces EMT-like changes in epithelial cells (13, 14). However, how epithelial cells regulate immune responses via these signaling pathways remains unclear and warrants further investigation.

Recent technological breakthroughs, notably single-cell RNA sequencing and spatial transcriptomics, have provided powerful tools to dissect the complex cellular dynamics in lung tissue after SiO_2_ exposure. In this study, we aim to elucidate the cellular and molecular mechanisms underlying SiO_2_-induced lung injury, with a particular focus on epithelial cells and their interactions with immune cells. Leveraging advanced approaches such as single-cell RNA sequencing and spatial transcriptomics, we sought to track epithelial cell dynamics and gene expression alterations following chronic SiO_2_ exposure. We hypothesized that chronic SiO_2_-induced epithelial injury could lead to the emergence of novel epithelial subsets that undergo partial EMT, while orchestrating inflammation and fibrosis through direct interactions with immune cells.

These findings shed light on a new epithelial-immune mechanism in SiO_2_-induced lung injury, highlighting the pivotal role of C0 cells in orchestrating immune responses and contributing to disease progression. Given the persistent global health burden of silicosis, particularly in highly industrialized and developing regions, insights into these mechanisms could pave the way for novel therapeutic strategies targeting epithelial–immune cell interactions in fibrotic lung diseases.

## 2 Materials and methods

### 2.1 Preprocessing of single-cell transcriptomes

Single-cell RNA sequencing data were obtained in matrix format from the Gene Expression Omnibus (GEO) under accession number GSE183682. Lung tissues were harvested from mice of normal saline (NS) and SiO_2_ groups at 7 days and 56 days (mouse and modeling methods as described in Chen et al.), immediately frozen in OCT on dry ice, and stored at -80°C before further processing (15). Quality control was performed using Seurat and DoubletFinder to identify and remove multiplets and low-quality cells. The Seurat package was also used to filter out foreign cells, where a gene was considered expressed if detected in more than 3 cells, and each cell was required to have at least 200 expressed genes. Following these quality control procedures, a total of 36,122 high-quality single cells were retained for downstream analysis. Gene expression data were subsequently normalized using Seurat’s “LogNormalize” method to reduce variability in gene expression counts.

### 2.2 Dimensionality reduction, clustering, and annotation

To identify highly variable genes (HVGs), Seurat’s “FindVariableFeatures” function was applied with default parameters. Principal component analysis (PCA) was then performed on the HVGs, and the top 30 principal components were retained for subsequent analysis. Uniform manifold approximation and projection (UMAP) was used to reduce the dimensionality of the data and to visualize the cellular clusters in two dimensions. Clustering of the cells was performed using Seurat’s “FindClusters” function. To correct for potential batch effects, the Harmony R package was employed to integrate datasets across different conditions.

### 2.3 Intracellular crosstalk analysis

Cell-cell interactions were analyzed using the CellChat R package, which relies on known ligand-receptor pairs to explore intercellular communication. The normalized counts of the merged samples were pre-processed using the identifyOverExpressedGenes, identifyOverExpressedInteractions, and projectData functions from the CellChatDB.human database. To identify significant ligand-receptor pairs, key CellChat functions, including computeCommunProb, computeCommunProbPathway, and aggregationNet, were applied. Interaction networks were visualized using CellChat’s netVisual_bubble function to assess the strength and significance of the interactions.

### 2.4 Differential expression and enrichment analyses

The “FindMarkers” function in Seurat was used to identify differentially expressed genes (DEG) in specific cell subgroups, with the following cutoff criteria: an average log fold change (Avg_logFC) greater than 0.25 and an adjusted p-value (p_val_adj) less than 0.05. To provide further biological insights into the DEGs, functional enrichment analysis was conducted using the ClusterProfiler R package to perform Gene Ontology (GO) analyses.

### 2.5 Trajectory analysis

Trajectory analysis was conducted using the Monocle 2.0 R package to investigate the differentiation processes of epithelial cells. The “differentialGeneTest” function was used to identify genes associated with pseudotime.

### 2.6 CellTrek analysis

To further explore the spatial transcriptomic data in conjunction with single-cell RNA-seq, the feature-barcode matrix was downloaded from GEO (accession number GSE183683). The “traint” function in the CellTrek R package was applied to co-embed the spatial transcriptomics (ST) data with the single-cell RNA-seq data. Subsequently, the celltrek function, using default parameters, was employed to project single cells onto the ST coordinates.

### 2.7 EMT score calculation

To score EMT related gene sets of each single cell in the scRNA-Seq data, the ‘AddModuleScore’ method from the Seurat package was utilized. Scores were calculated by expressions of EMT related genes noted in result section.

### 2.8 Animal models and ethical compliance

Male C57BL/6 mice (8–12 weeks; body weight range 20–24 g) were purchased from Cavion Experimental Animal Co., LTD., Changzhou City, Jiangsu Province, China (animal license number SCXY (Su) 2011-0003). Animals were raised under the condition of no specific pathogen (SPF), with a temperature of 22-24 °C, relative humidity of 50 ± 5%, and a day-night cycle of 12 hours of light and 12 hours of darkness (lights at 08:00). After a 7–14 day adaptation period, all mice were randomly divided into two groups of 12 mice each: the NS group (NS) and the silica exposure group (SiO_2_).

The mice were then anesthetized by inhaling isoflurane and intranasal instillation of silica (20ug/ul, 80ul) diluted in saline was used (SiO_2_). The animals in the NS group were given the same dose of normal saline nasal drops, and the mice were sacrificed at the corresponding time points, and then the lung tissues were collected and stored for further experiments.

All procedures followed the Guide for the Care and Use of Laboratory Animals (NIH Publication No. 8023, revised 1978) and were approved by the Institutional Animal Care and Ethics Committee of Anhui University of Science and Technology.

### 2.9 Immunohistochemistry and immunofluorescence staining

For immunohistochemistry (IHC) staining, paraffin-embedded lung tissue sections were first cleared of paraffin with xylene and rehydrated in a series of ethanol dilutions. To expose antigens, sections were heated in Citrate Antigen Retrieval Solution (Beyotime, P0090) for 30 minutes. Endogenous peroxidase activity was blocked by treating the slides with 3% hydrogen peroxide for 10 minutes. Permeabilization was conducted with 0.3% Triton^®^ X-100 (BioFroxx, 1139) at room temperature for 10 minutes. After a 1-hour block with 3% BSA-PBST, primary antibodies were applied and left on the sections overnight at 4°C. The following day, HRP-linked secondary antibodies were introduced for 1 hour at room temperature, and staining was developed using 3,3’-diaminobenzidine (DAB). Hematoxylin was used to stain nuclei, after which sections were dehydrated, coverslipped, and examined under a light microscope.

For immunofluorescence staining, paraffin sections of mice lungs were dewaxed, rehydrated in a series of ethanol dilutions, immersion in hydrogen peroxide, repairing antigen, permeabilization, and then blocking for 1 hour in 10% goat serum at room temperature. Subsequently, the primary antibody was incubated overnight in a 4°C refrigerator after staining, rewarmed at room temperature, and then stained with the secondary antibody at room temperature for 1 hour. Finally, nuclei were stained with DAPI (1:1000) for 10 minutes. All tissue sections were mounted on glass slides and covered with an anti-fade mounting medium. Images were captured using a confocal microscope (Olympus, FV-3000). The antibodies used in this experiment are listed in **Table 1**.

**Table 1:** Details of antibodies used in IHC and IF experiments.

Antibody	Host	Dilution ratio	Company
SPP1	Rabbit	1:200-800	CST,88742s
Sftpc	Rabbit	1:50-500	Proteintech,10774-1-AP
CD206	Rabbit	1:400-800	CST,24595s
DAPI		1:1000	Beyotime, C1002

### 2.10 Statistical analysis

Data are presented as mean ± standard error of the mean (SEM). Homogeneity of variances across groups was examined using the Brown-Forsythe and Bartlett’s tests. Comparisons involving two groups were performed using an independent-samples t-test. For comparisons involving three or more groups, two-way ANOVA was conducted, followed by Bonferroni’s multiple comparisons test. GraphPad Prism 9.0.0 software was used to perform the statistical analyses, where p< 0.05 was set as the level of significance for determining any significant differences.

## 3 Results

### 3.1 Marked epithelial cell proliferation at Day 56 upon SiO_2_ treatment

**Figure 1:**
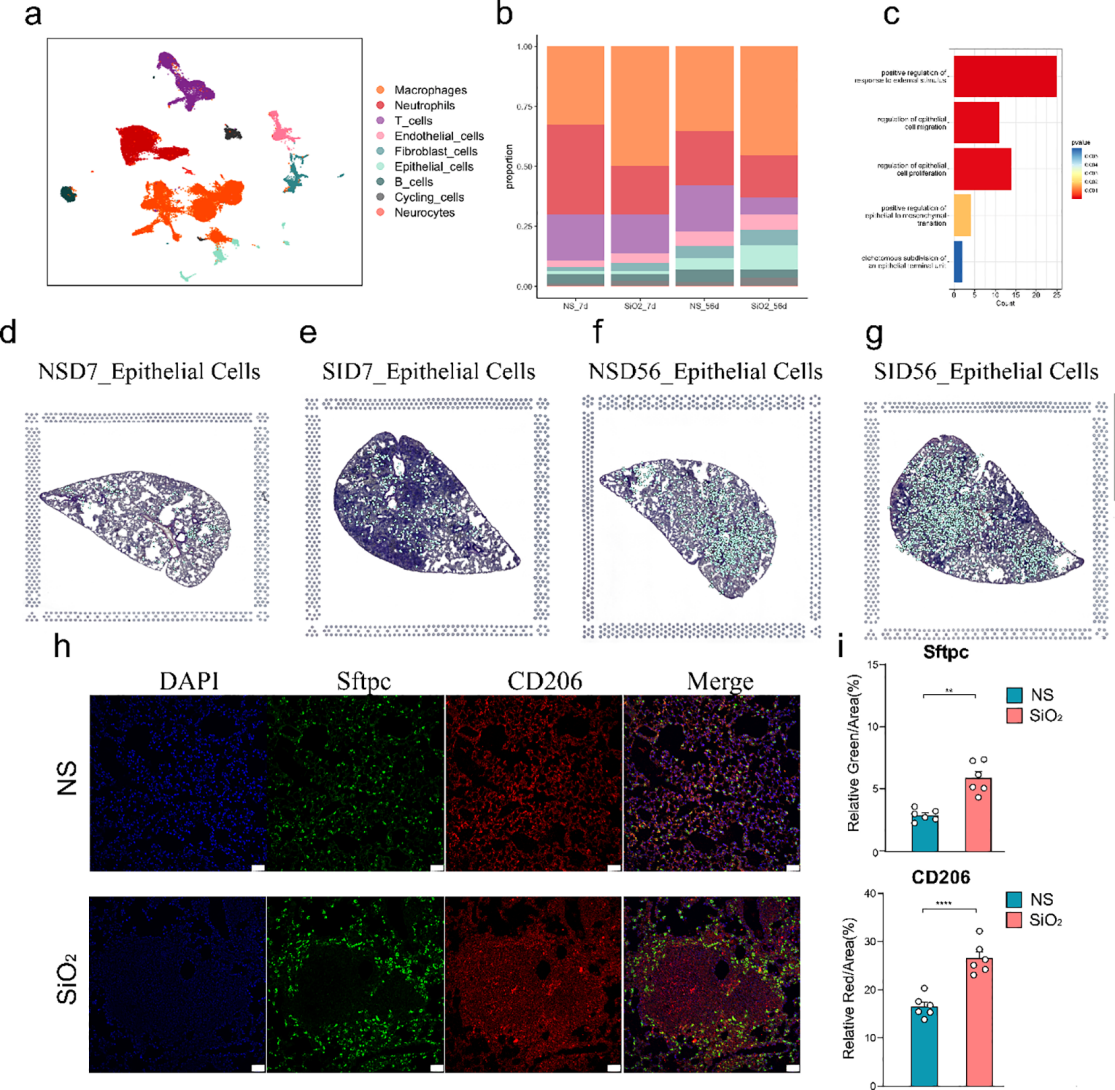
Single-cell RNA sequencing analysis of lung tissue in SiO_2_-induced injury. **(a)** UMAP plot showing the distribution of different cell types across various treatment groups (NS_7d, SiO_2__7d, NS_56d, SiO_2__56d). **(b)** The proportion of different cell types in each treatment group. Epithelial cell percentages are as follows: NS_7d (1.23%), SiO_2__7d (1.25%), NS_56d (8.00%), SiO_2__56d (10.00%). **(c)** GO enrichment analysis of differentially expressed genes in epithelial cells. **(d-g)** UMAP plots (left) and spatial distribution of epithelial cells (right) for each group at 7 and 56 days post-treatment: NS_7d **(d)**, SiO_2__7d **(e)**, NS_56d **(f)**, and SiO_2__56d **(g)**. **(h, i)** Immunofluorescence staining and quantification of Sftpc (green) and CD206 (red) in NS and SiO_2_ group lung tissues, with DAPI (blue) nuclear staining. Significance levels: P **** p ≤ 0.0001; P *** p ≤ 0.001; P ** p ≤ 0.01; P * p≤ 0.05; NS P>0.05.

To investigate the effects of SiO_2_ exposure on lung tissues, we first performed dimensionality reduction using UMAP to visualize the distribution of different cell types across various experimental groups (NS_7d, SiO_2__7d, NS_56d, SiO_2__56d) using data obtained from the GEO under accession number GSE183682 (**Figure 1a**). We utilized Seurat for preprocessing the single-cell RNA sequencing data, including quality control and normalization. UMAP plots revealed distinct cell clusters, the SiO_2_-treated group at 56 days exhibited markedly greater changes compared to other groups. In particular, significant alterations were observed not only within immune cell populations but also prominently among epithelial cells (**Figure 1b**). This marked epithelial cell response prompted us to further investigate their potential roles in mediating SiO_2_-induced lung injury.

The increased number of epithelial cells in the SiO_2_-treated group at 56 days suggests a possible involvement of cellular proliferation and repair processes induced by SiO_2_ exposure. GO enrichment analysis of differentially expressed genes in epithelial cells from the disease groups revealed significant upregulation of genes involved in epithelial cell migration, proliferation and responses to external stimuli (**Figure 1c**). These findings indicate that SiO_2_ exposure enhances epithelial cell expansion, likely through heightened proliferative and stress-responsive activities.

We then combined spatial transcriptomics with single-cell RNA sequencing, which revealed distinct patterns in the distribution and gene expression of various cell types across the tissue sections (**Supplementary Figure 1**). In the 7-day treatment groups, both NS and SiO_2_ exposures exhibited sparse distribution of epithelial cells across the tissue. By day 56, however, epithelial cells markedly increased in abundance and formed distinct clusters, especially within SiO_2_-exposed tissues (**Figures 1d-g**). Additionally, spatial analysis revealed that macrophages were more abundant in the SiO_2_-treated tissues at 56 days, which coincided with the increase in epithelial cells (**Supplementary Figure 1**).

To further validate these observations, we performed immunofluorescence staining on lung tissue sections from the NS_56d and SiO_2__56d groups (**Figures 1h-i**). In the NS_56d group, Sftpc^+^ epithelial cells (green) were evenly distributed with moderate intensity, accompanied by a sparse presence of CD206^+^ M2 macrophages (red). In contrast, the SiO_2__56d group exhibited a significant increase in Sftpc^+^ epithelial cells and CD206^+^ macrophages. Quantitative analysis confirmed that both Sftpc and CD206 signals were significantly elevated in the SiO_2__56d group compared to NS_56d. Interestingly, Sftpc-positive cells were found to be localized around CD206-positive macrophages in the SiO_2_-treated group, suggesting a coordinated expansion of epithelial and macrophage populations in response to SiO_2_-induced injury (**Figure 1h**).

Taken together, these results revealed that SiO_2_ exposure leads to significantly enhanced epithelial cell proliferation and a heightened immune response. These findings imply that SiO_2_ exposure may induce a complex tissue response, involving both immune activation and epithelial cell proliferation.

### 3.2 Spatial transcriptomics confirms late-stage expansion of C0 cells with elevated Sftpc and Ccl6

**Figure 2:**
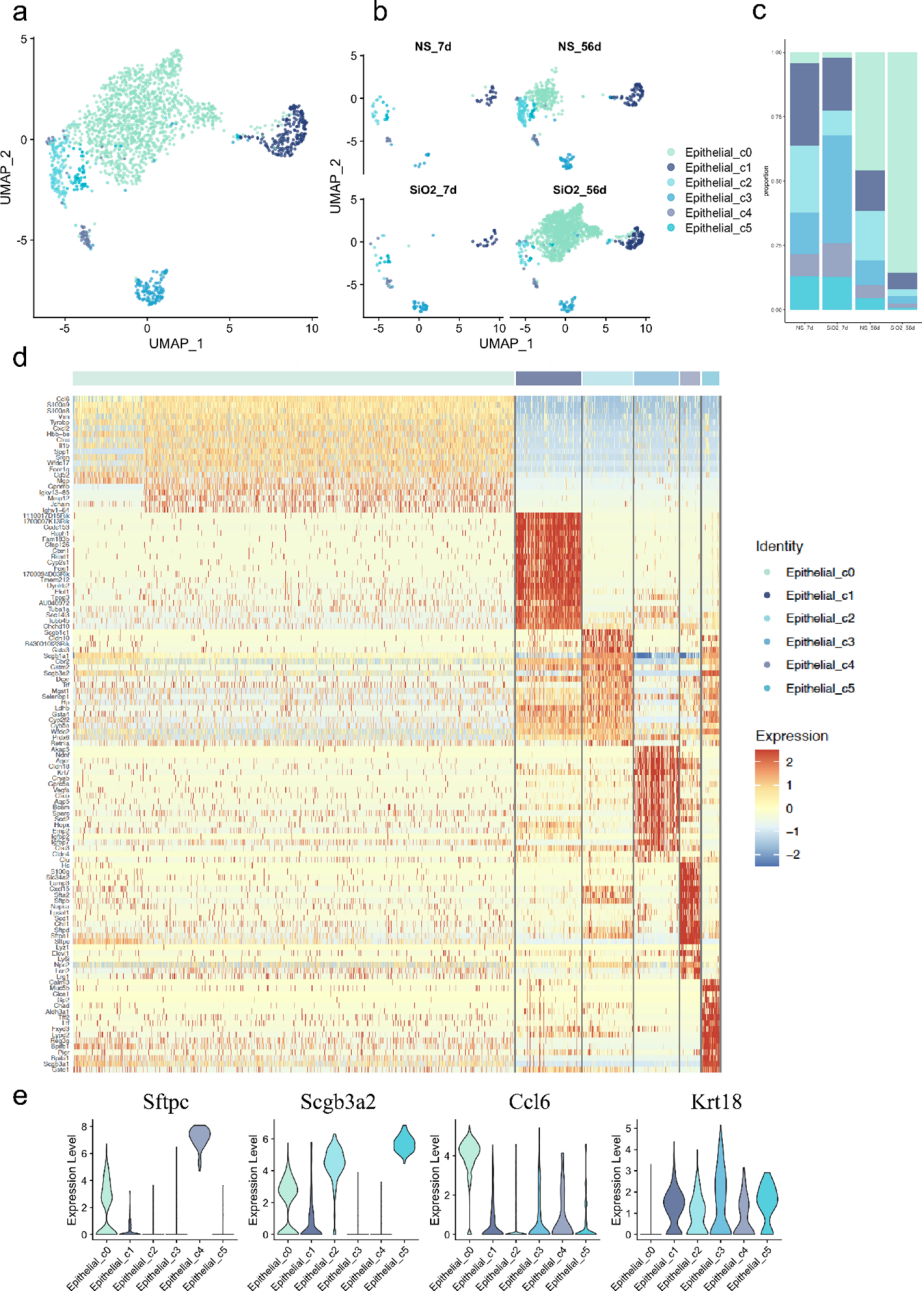
Characterization of epithelial subpopulations in SiO_2_-induced lung injury. **(a)** UMAP plot showing the distribution of epithelial cell subpopulations across all treatment groups. **(b)** UMAP plot focusing on the distribution of epithelial subpopulations (C0-C5) across NS_7d, SiO_2__7d, NS_56d, and SiO_2__56d groups. **(c)** The proportion of each epithelial subpopulation (C0-C5) in different treatment groups. **(d)** The expression of differentially expressed genes across epithelial subpopulations (C0-C5). **(e)** The expression levels of *Sftpc*, *Scgb3a2*, *Ccl6*, and *Krt18* across epithelial subpopulations (C0-C5).

Next, we aimed to investigate the relationship between different subclusters. The gene expression data show that the epithelial cells were classified into 5 distinct groups, with SiO_2_ exposure significantly altering their composition (**Figures 2a-c**). The UMAP analysis (**Figure 2b**) shows that at 7 days, the NS and SiO_2_ groups have similar distributions of epithelial subpopulation. However, we noticed C0 cells a marked shift in the SiO_2__56d group to dominate. This was further confirmed by the proportional distribution of epithelial subpopulations (**Figure 2c**), where C0 cells were significantly more abundant in the SiO_2__56d group compared to other groups.

Violin plots and heatmap analyses revealed distinct gene expression signatures for each cell cluster (**Figures 2d, e**). Cluster C1 cells, marked by high expression of *Foxj1* and *Tppp3*, were identified as ciliated airway epithelial cells. C2 cells exhibited elevated *Scgb1a1*, a canonical marker of secretory club cells. C3 cells exhibited co-expressing *Krt17* (progenitor/stress marker) and *Ager* (AT1 marker), suggesting that this population may represent transitional epithelial cells committed toward the AT1 lineage (16). C3 cells also expressed *Cldn18*, *Cldn4*, and *Bcam*, indicating roles in tight junction formation and barrier maintenance within the respiratory epithelium. C4 cells were identified as *Lamp3* ^+^ and *Sftpc* ^+^ AT2 cells. C4 cells also expressed innate immune response genes, including *Lyz1*, *Lyz6*, *Cxcl15*, and *Sfta2*. Lastly, C5 cells, another secretory population, displayed high levels of *Scgb3a2* and *Scgb3a1*, along with *Calml3*, *Chad*, and *Aldh3a1*, suggesting functions in epithelial cell development and response to wounding.

In contrast, C0 cells expressed epithelial markers such as *Scgb3a2* and *Sftpc* but were distinguished by EMT-related genes, including *Spp1*, *Mmp12*, *Ctss*, and *Vim*. Unlike other clusters, C0 cells lacked *Krt18*, a marker of epithelial differentiation expressed across all other subpopulations, suggesting a mesenchymal transition (17–19). Further analysis revealed high expression of inflammatory activation markers *S100a8* and *S100a9*, alongside immune- and inflammation-related genes such as *Ccl6*, *Cxcl2*, and *Il1b*. These genes, linked to chemotaxis and immune cell recruitment (5, 20–22), collectively indicate that C0 cells actively contribute to immune modulation and inflammatory responses in the SiO_2__56d group.

**Figure 3:**
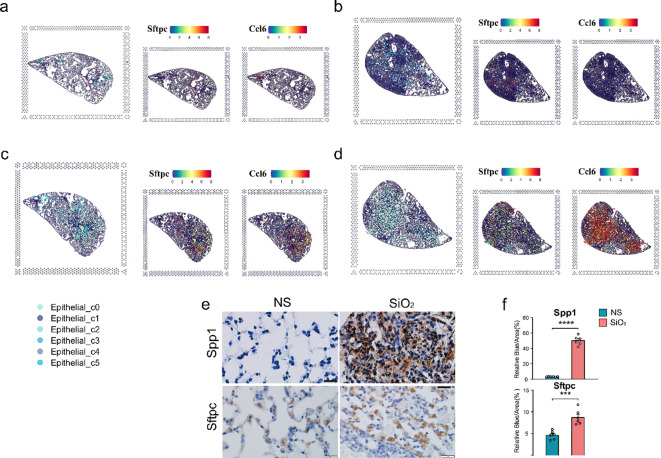
Spatial distribution of epithelial subpopulations in SiO_2_-induced lung injury. **(a-d)** Spatial expression of *Sftpc* (left) and *Ccl6* (right) in epithelial subpopulations (C0-C5) across different time points: **(a)** NS_7d, **(b)** SiO_2__7d, **(c)** NS_56d, and **(d)** SiO_2__56d. **(e, f)** Immunohistochemistry staining and quantification of Spp1 (brown) and Sftpc (brown) in NS and SiO_2_-treated lung tissues. P **** p ≤ 0.0001; P *** p ≤ 0.001.

To map these dynamics over time, we employed spatial transcriptomics. At 7 days post-exposure, both the NS and SiO_2_ groups exhibited sparse C0 cell populations (**Figures 3a, b**). By 56 days, however, C0 cells were significantly more abundant in the SiO_2_ group (**Figures 3c, d**). To assess C0 cell dynamics, we examined the spatial expression of *Sftpc* and *Ccl6* (**Figure 3c**). SiO_2__56d tissue showed elevated and localized *Sftpc* expression, while *Ccl6* expression was markedly elevated and widespread (**Figure 3d**). Immunohistochemical staining further confirmed these findings (**Figures 3e, f**). In SiO_2__56d lungs, *Spp1* expression was markedly elevated, co-occurring with a significant increase in *Sftpc*-positive cells compared to controls. Quantitative analysis showed significant increases in both markers’ area percentages, consistent with a partial EMT and inflammatory signatures in SiO_2_-induced injury.

Together, these findings illuminate the dynamic epithelial response to SiO_2_ exposure. The C0 cell population expresses epithelial markers such as *Sftpc* and *Scgb3a2* but is distinguished by co-expression of EMT-related genes like *Mmp12* and *Vim*, indicating an ongoing transition toward a mesenchymal phenotype. Notably, C0 cells also upregulate a suite of immune-related genes—including *Ccl6*, *S100a8*, and *Cxcl2*—driving immune cell recruitment and amplifying inflammatory responses. In contrast, other clusters maintain distinct epithelial identities while contributing to cellular defense. For instance, the *Lamp3* ^+^ and *Sftpc* ^+^ AT2 cells (C4) express innate immune response genes such as *Lyz1* and *Cxcl15*, supporting antimicrobial defense and tissue protection.

### 3.3 Pseudotime analysis reveals C0 emergence from C1

**Figure 4:**
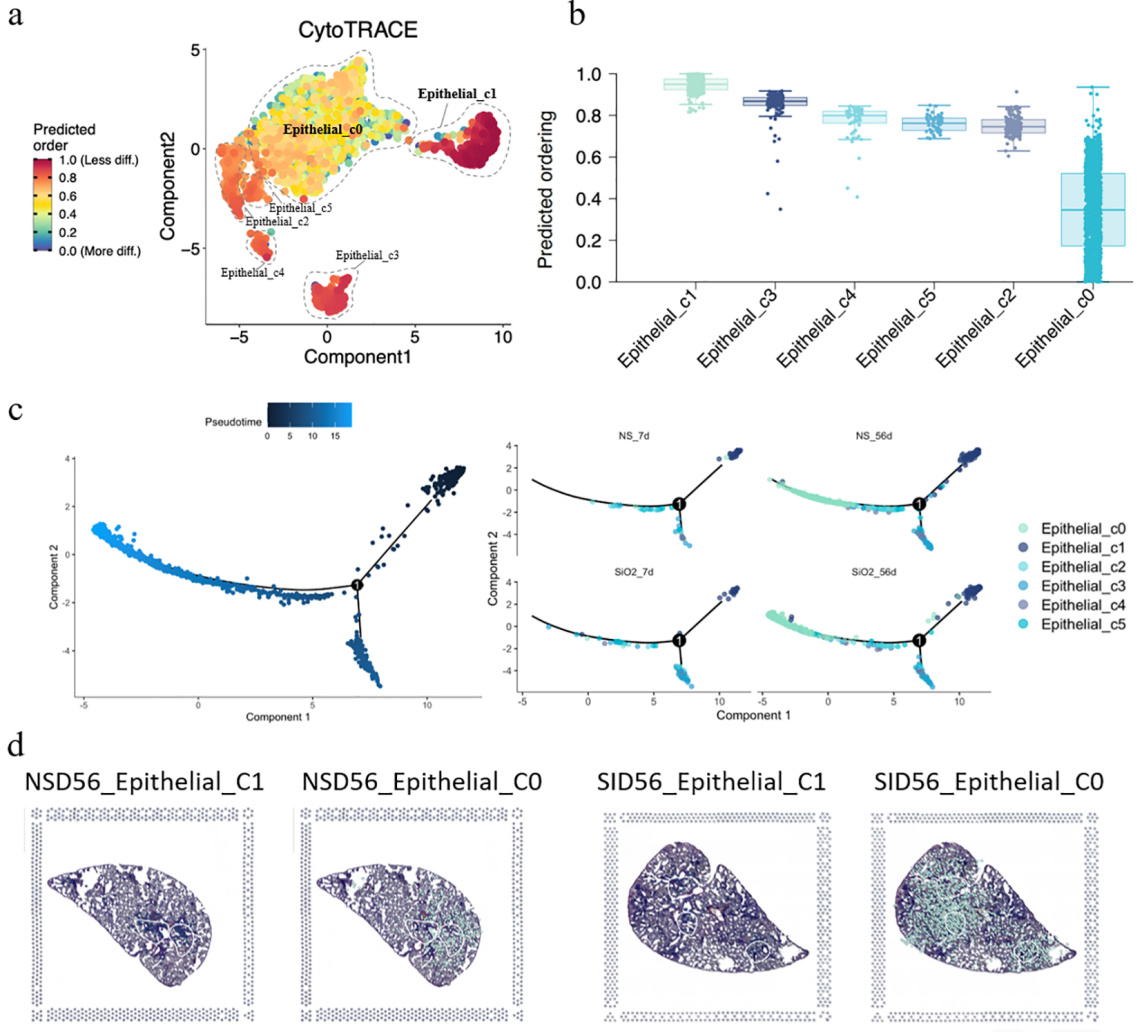
Differentiation and trajectory of epithelial subpopulations in SiO_2_-induced lung injury. **(a)** CytoTRACE analysis predicting the differentiation potential of epithelial subpopulations (C0-C5). **(b)** The predicted ordering and differentiation potential across different epithelial subpopulations. **(c)** Pseudotime trajectory analysis of epithelial subpopulations at different time points (NS_7d, SiO_2__7d, NS_56d, SiO_2__56d) showing the progression of differentiation from C1 to C0 cells. **(d)** Spatial expression of epithelial subpopulations (C0, C1) in NS_56d and SiO_2__56d lung tissues.

Having established the expansion and phenotypic characteristics of C0 cells, we next sought to investigate their developmental origin. To achieve this, we employed CytoTRACE analysis to assess the differentiation potential of epithelial cell subpopulations. The CytoTRACE analysis plot reveals that C0 cells are at the final stage of differentiation, representing mature or post-differentiation cells, while C1 and other cells are at an earlier, progenitor-like stage (**Figure 4a**). The stemness score shows that C1 cells exhibit the highest stemness score, with C0 the lowest (**Figure 4b**). This suggests that C1 cells may be involved in early-stage differentiation or progenitor functions, while C0 cells likely participate in later stages of differentiation or mature epithelial functions. This is further supported by pseudotime analysis of the epithelial cells, both with and without SiO_2_ treatment (**Figure 4c**). For this analysis, we selected C1 cells as the pseudotime starting point, revealing that C0 cells represent the most differentiated state at the end of the trajectory. These findings align with our earlier observation that *Krt18* was absent in C0 cells, further suggesting that they occupy a more advanced and differentiated state.

Next, pseudotime trajectory analysis and differential expression analysis revealed a clear distinction between C0 and C1 cells, as visualized in the pseudotime heatmap (**Supplementary Figure 2**) and volcano plot (**Supplementary Figure 4**). C0 cells were characterized by high expression of immune response genes, such as *Ccl6*, *S100a8*, *S100a9*, and *Il 1b*, which are involved in inflammation and immune signaling. Additionally, C0 cells exhibited elevated levels of EMT markers such as *Spp1* and *Vim*, suggesting their active involvement in tissue repair or remodeling. Over the pseudotime trajectory, we observed significant downregulation of genes associated with epithelial functions, including tight junction components (*Cldn4*, *Cldn18*), an undifferentiated epithelial marker (*Krt7*), canonical surfactant genes (*Sftpa1*, *Sftpb*, *Sftpd*), and secretory epithelial markers (*Scgb1a1*, *Scgb3a1*, *Scgb3a2*). These shifts indicate that C0 cells diverge from traditional epithelial roles, reinforcing their specialization in immune modulation and mesenchymal transition during SiO_2_-induced injury.

To further explore the spatial distribution of C0 and C1 cells, we performed spatial transcriptomics on the NS_56d and SiO_2__56d groups. Intriguingly, the data showed that C0 and C1 cells are consistently juxtaposed in both treatment groups, regardless of SiO_2_ exposure (**Figures 3c, d**). This spatial proximity, coupled with pseudotime trajectory analysis (**Figure 4d**), suggests a potential developmental relationship between these clusters. Specifically, C0 cells exhibit elevated expression of EMT markers like *Vim, Spp1*, and *Mmp12*, while C1 cells retain ciliated epithelial traits marked by *Foxj1*, hinting that C0 cells may differentiate from C1 cells along a trajectory involving mesenchymal transition. We propose that under SiO_2_-induced stress, C0 cells emerge from C1 precursors and subsequently undergo partial EMT, as evidenced by their upregulation of immune genes (*Ccl6*, *S100a8*) and EMT markers, enabling their migration and expansion to support tissue remodeling and inflammation in response to chronic injury.

### 3.4 C0 cells exhibit increased immune interactions and upregulation of key ligand–receptor interactions in silica-injured lung

**Figure 5:**
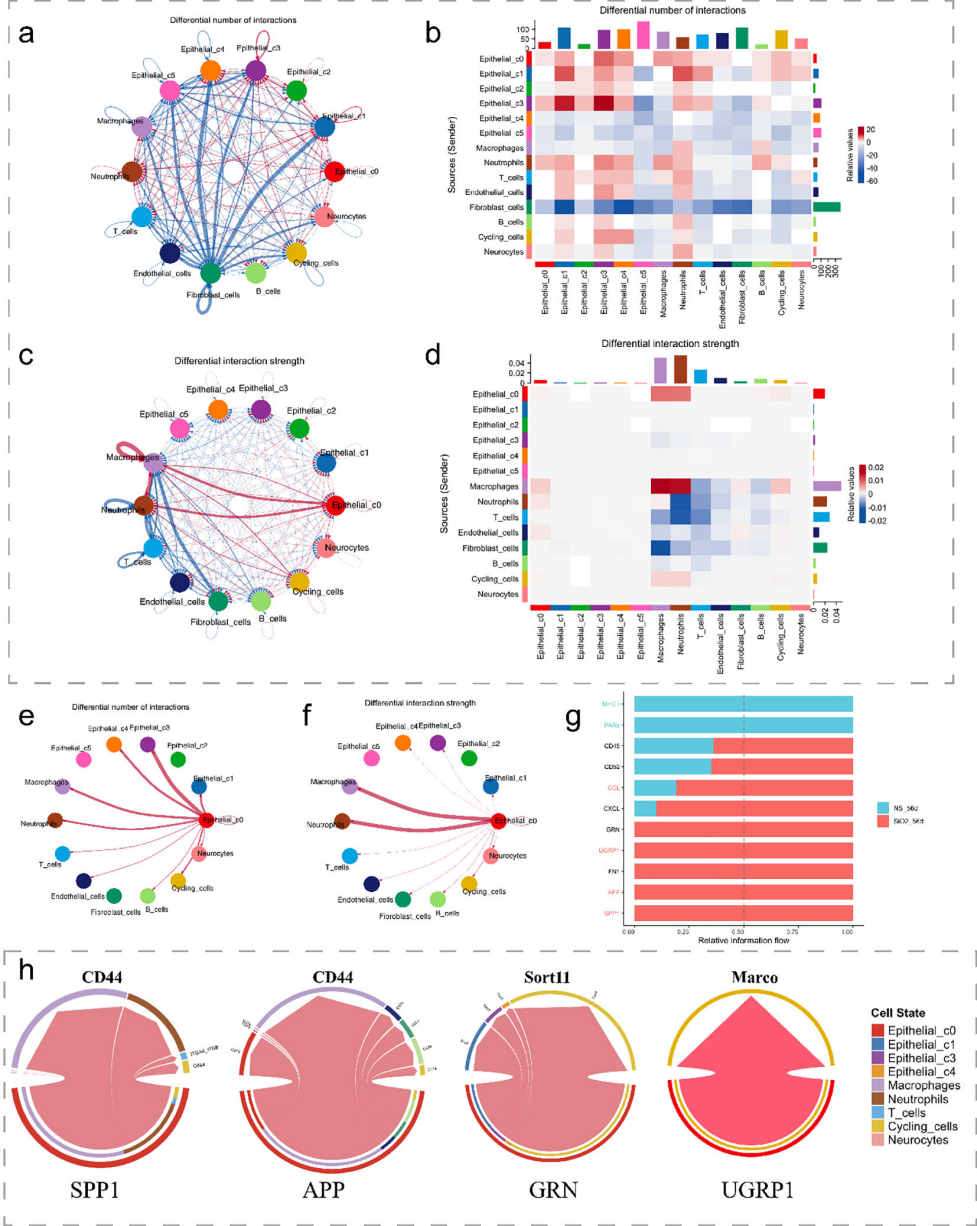
Cell-cell interactions in SiO_2_-induced lung injury. **(a, b)** The differential number of interactions between epithelial subpopulations and other cell types. **(c, d)** The differential interaction strength between epithelial subpopulations and other cell types. **(e, f)** Interaction network of C0 cells with macrophages and other immune cells in SiO_2_-treated tissues. **(g)** Relative information flow between signaling interactions in the SiO_2_-treated group. **(h)** The differential signaling interactions (SPP1, APP, GRN, and SCGB3A2) and their interactions with different cell types.

We next investigate the signaling pathways and intercellular communication profiles of C0 cells. Firstly, using the CellChat analysis, we identified a substantial increase in both the number and strength of cell-cell interactions in the SiO_2__56d group compared to the NS_56d group. C0 cells in the disease group exhibited significantly enhanced interaction strength and a greater number of interactions with immune cells, particularly macrophages and neutrophils, with a smaller portion directed towards cycling cells (**Figures 5a–d**).

Further analysis of ligand-receptor interactions showed a marked increase in relative information flow for APP, SPP1, SCGB3A2, and GRN signaling in the SiO_2__56d group (**Figures 5e–g**). These pathways were categorized by their target cells: SPP1 and APP predominantly engaged macrophages and neutrophils, while SCGB3A2 and GRN mainly interacted with cycling cells (**Figure 5h**). Expression of SPP1 and APP correlated with pro-inflammatory responses, while SCGB3A2 and GRN aligned with anti-inflammatory and proliferative functions (1, 5, 23). These patterns suggest C0 contributes to both immune activation and tissue repair in SiO_2_-induced injury.

**Figure 6:**
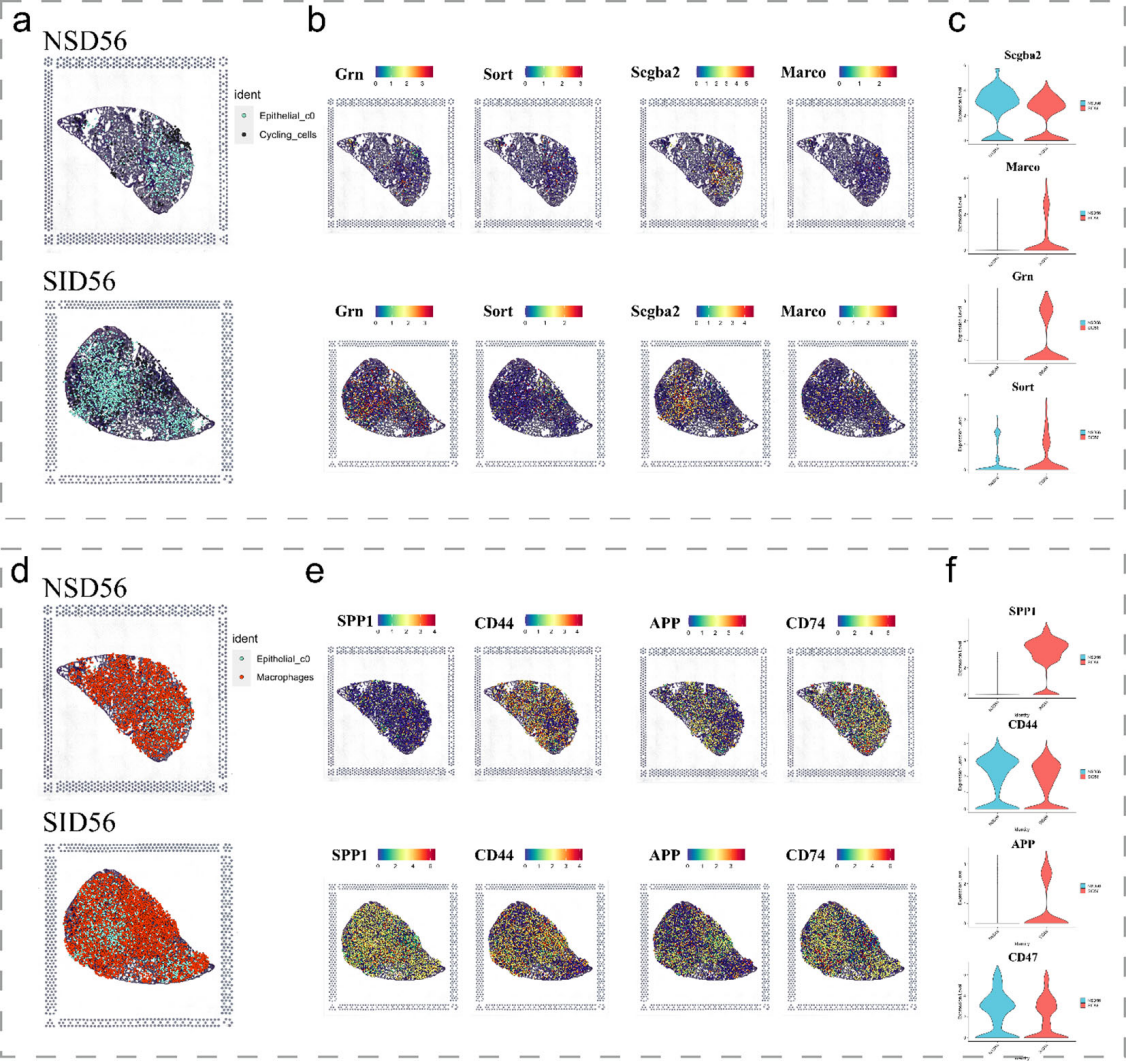
Spatial expression of key immune and epithelial markers in SiO_2_-induced lung injury. **(a-c)** Spatial expression of immune markers and epithelial markers in NS_56d and SiO_2__56d lung tissues, visualized with spatial transcriptomics. **(d-f)** Spatial expression of immune-related markers in NS_56d and SiO_2__56d lung tissues.

Subsequent analysis of spatial transcriptomics data between cycling cells and C0 cells (**Figures 6a-c**) revealed that, as expected, *Scgb3a2* and *Grn* were predominantly expressed in C0 cells, while their corresponding receptors, *Sort1* and *Marco*, exhibited moderate degree of expression in cycling cells. Notably, both pairs of ligands and receptors were significantly increased in the disease group. Similarly, the ligands SPP1 and APP, along with the receptors CD44 and CD74, were observed to be expressed in C0 cells and macrophage populations, respectively (**Figures 6d-f**). Furthermore, ligand genes *Spp1* and *App* were significantly upregulated in the SiO_2__56d samples compared to the NS_56d groups (**Figures 6d-f**), while their ligands, CD44 and CD74, showed a milder upregulation. Additionally, spatial transcriptomics data highlighted a significant rise in CD44 expression in neutrophils (**Supplementary Figure 3**). Collectively, these findings indicate that C0 cells amplify ligand-driven signaling, via SCGB3A2, SORT, SPP1 and APP, in SiO_2_-induced injury, likely enhancing immune cell recruitment and tissue repair through interactions with macrophages and cycling cells.

### 3.5 Partial EMT dynamics during C1 to C0 transition are enhanced by neutrophil-derived signaling

Previous analyses revealed shifts in epithelial cell populations, with C0 cells emerging as key mediators of immune responses and tissue remodeling after SiO_2_ exposure. Interestingly, these cells exhibit mesenchymal traits, suggesting EMT. To explore this, we analyzed EMT-related gene expression along the pseudotime trajectory.

**Figure 7:**
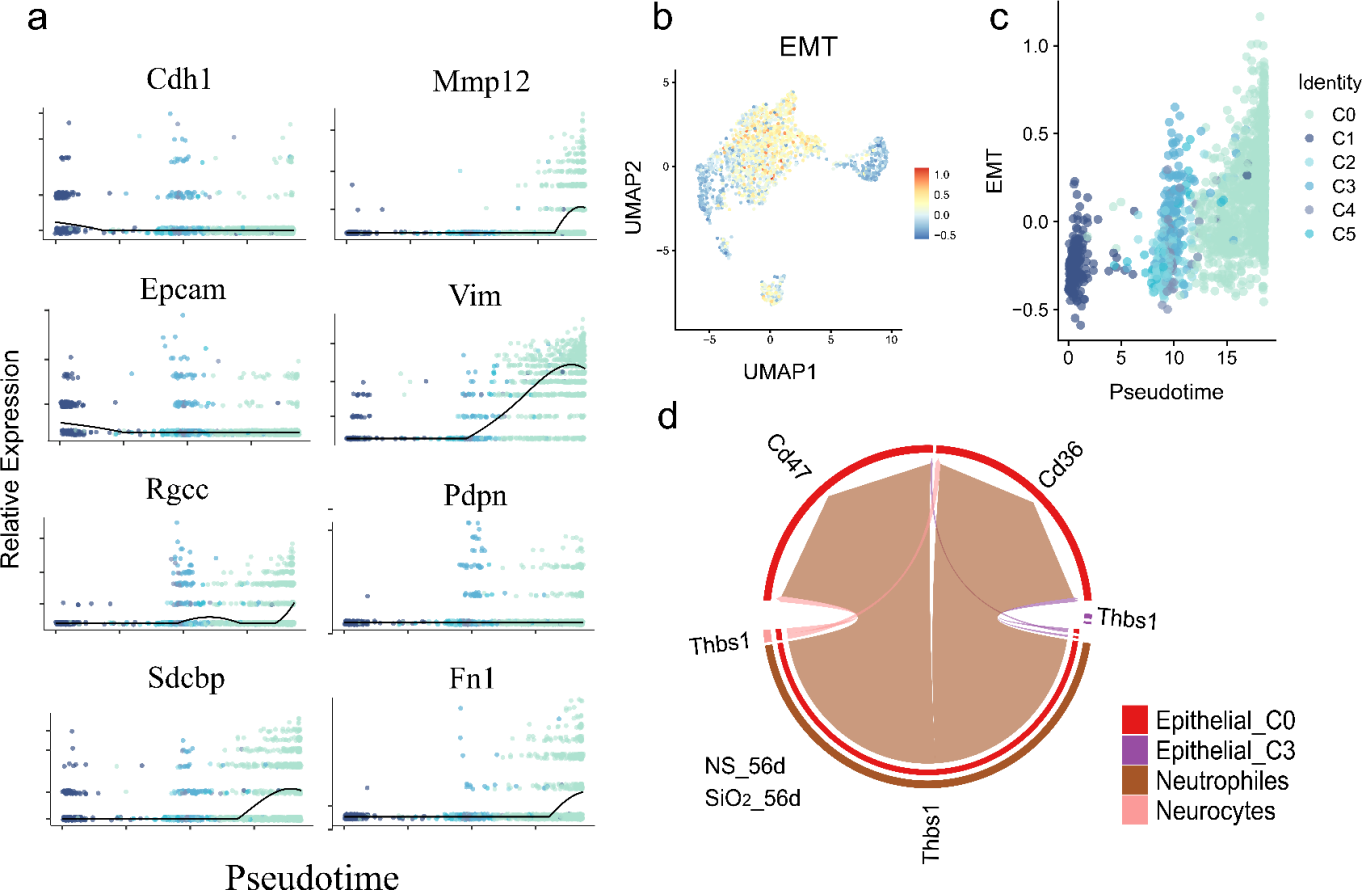
Partial EMT gene expression dynamics and neutrophil-epithelial interactions during pseudotime differentiation. **(a)** Temporal expression patterns of key EMT-related genes along pseudotime, showing significant upregulation of mesenchymal markers (*Vim*, *Fn1*, *Zeb1*, *Dab2* etc.) and downregulation of epithelial markers (*Epcam*, *Cdh1*). **(b)** Quantitative EMT index plotted across pseudotime. **(c)** UMAP visualization of EMT index distribution. **(d)** CellChat analysis of ligand-receptor interactions.

We observed distinct temporal patterns in EMT markers. Mesenchymal genes, including *Vim*, *Fn1*, *Zeb1*, and *Dab2*, were upregulated, while canonical epithelial markers *Epcam* and *Cdh1* were downregulated as cells progressed toward the C0 phenotype (**Figure 7a**). These changes peaked in mature C0 cells, indicating a progressive but incomplete EMT. Since C0 cells also expressed differentiated epithelial markers like *Sftpc* and *Scgb3a2*, this suggests partial EMT rather than a full mesenchymal transition.

To quantify EMT dynamics, we used the AddModuleScore function to calculate an EMT index, which increased along the pseudotime trajectory, confirming a partial EMT from C1 progenitors to C0 cells (**Figure 7b**). UMAP visualization showed elevated EMT scores in C0 cells, confirming their distinct mesenchymal-like phenotype (**Figure 7c**).

To investigate drivers of this process, we examined cell-cell interactions using CellChat. Neutrophils exhibited strong ligand-receptor communication with C0 cells via neutrophil-derived ThBS1 interacting with Cd47 and Cd36 receptors on C0 cells (**Figure 7d**). ThBS1 is known to promote EMT and epithelial plasticity in cancer and tissue regeneration (24, 25).

These findings propose a model where neutrophil-derived ThBS1 drives partial EMT in epithelial cells, promoting differentiation of C1 progenitors into inflammatory, mesenchymal-like C0 cells that retain epithelial markers. This immune-epithelial crosstalk underscores epithelial-mesenchymal plasticity as a dynamic process in SiO_2_-induced lung remodeling.

## 5 Discussion

In this study, we utilized single-cell RNA sequencing and spatial transcriptomics to characterize the temporal and spatial dynamics of lung epithelial cells following SiO_2_ exposure. Our analyses identified a distinct epithelial subset, C0 cells, which expanded significantly by day 56 post-exposure. These cells co-expressed alveolar cell markers (*Sftpc*, *Scgb3a2*) with mesenchymal transition and immune-related genes (*Spp1*, *Mmp12*), yet lacked *Krt18*, suggesting a shift from traditional epithelial differentiation. These findings position C0 cells as key contributors to immune responses and tissue remodeling in SiO_2_-induced lung injury.

C0 cells expressed a robust set of chemokines and cytokines (*Ccl6*, *S100a8*, *S100a9*), alongside CellChat analyses showing enhanced interactions with macrophages and neutrophils. This expression profile, supported by spatial co-localization with CD206^+^ macrophages (**Figure 1h**), underscores C0’s role in driving the recruitment and infiltration of immune cells in response to SiO_2_-induced injury. Pseudotime analysis further revealed that C0 cells likely emerge from *Foxj1^+^* C1 precursors, downregulating epithelial markers (*Krt7*, *Cldn4*, *Scgb1a1*) while upregulating EMT genes (*Spp1*, *Vim*, *Mmp12*). These shifts, validated by immunohistochemistry showing elevated Spp1 and Sftpc in SiO_2__56d lungs (**Figures 3e-f**), suggest that C0 cells undergo partial EMT and enhance their immunomodulatory capacities. Such plasticity may also amplify inflammation and contribute to fibrotic repairment.

Furthermore, our detailed analysis of EMT-related gene expression dynamics along the pseudotime trajectory (Section 3.5) demonstrated upregulation of mesenchymal genes like *Vim*, *Fn1*, and *Zeb1*, as well as downregulation of epithelial markers such as *Epcam* and *Cdh1*. Intriguingly, cell-cell interaction analyses identified neutrophil-derived *ThBS1* as a critical signaling molecule potentially driving this partial EMT via its receptors Cd47 and Cd36 expressed on C0 cells (**Figure 7d**). This finding aligns with Nickel et al.’s results that ThBS1 modulates TGF-β signaling to regulate EMT, further supporting Thbs1’s key role in driving epithelial plasticity across different biological contexts (24).

Signaling pathway analysis highlighted key ligand-receptor pairs mediating C0-immune interactions, including SPP1-CD44, APP-CD74, SCGB3A2-SORT1, and GRN-MARCO. In SiO_2__56d, *Spp1* and *App* were markedly upregulated in C0 cells (**Figures 6d-f**), driving interactions with macrophages and neutrophils, while *Scgb3a2* and *Grn* targeted cycling cells, consistent with their respective pro-inflammatory and repair roles. The SPP1-CD44 axis, previously linked to neutrophil activation in other contexts, may similarly promote immune infiltration here. These networks suggest C0 cells coordinate a dual response, balancing inflammation and repair, though functional assays are needed to confirm these effects.

The hybrid phenotype of C0 cells, combining epithelial and mesenchymal features, echoes transitional epithelial states reported in lung injury models. Choi et al. (26) described damage-associated transient progenitors derived from AT2 cells, which exhibit immune-responsive traits (26). Similarly, Kobayashi et al. (27) identified a pre-alveolar type-1 transitional state with a partial epithelial-mesenchymal phenotype during fibrosis (27). Like these states, C0 cells lack *Krt18* and upregulate *Spp1* and *Vim*, serving as an inflammation-associated epithelial subtype under SiO_2_ exposure. This convergence suggests that C0 cells may represent a conserved response to chronic lung stress, distinct from the canonical AT2-to-AT1 differentiation pathway.

However, an unresolved question is why these C0 cells maintain or even upregulate epithelial markers such as *Sftpc* and *Scgb3a2* despite their mesenchymal background. Our analyses offer potential insights into this paradox through identifying candidate upstream signaling mechanisms. First, enhanced ligand–receptor interactions involving SCGB3A2 and its receptor SORT1 (**Figures 5g-h**, **Figures 6a-c**) suggest that SCGB3A2 may exert autocrine or paracrine signaling to maintain epithelial gene expression within cells undergoing EMT-like transitions (28). Second, the observed neutrophil-derived ThBS1 signaling via receptors Cd47 and Cd36 expressed on C0 cells (**Figure 7d**) indicates that neutrophil-mediated signaling pathways might further drive this epithelial-mesenchymal plasticity. These findings highlight the complexity of signaling interactions influencing epithelial marker retention despite an ongoing mesenchymal transition, warranting further experimental validation.

Despite these insights, several limitations must be addressed. The lack of *in vivo* lineage tracing or C0-specific depletion limits our ability to definitively confirm their origin from C1 cells or their direct impact on fibrosis progression. Additionally, reliance on mouse models and omics data raises questions about C0’s relevance to human silicosis, and whether their emergence is reversible remains unexplored. Future studies should employ targeted interventions and extend analyses to human samples to address these gaps.

In conclusion, we identified C0 cells as a novel epithelial subset in SiO_2_-induced lung injury, marked by a hybrid epithelial-immune-mesenchymal phenotype. Their expansion, EMT-like features, and immune interactions highlight epithelial plasticity as a driver of inflammation and remodeling in silicosis. Targeting C0-mediated pathways, such as SPP1-CD44, may offer therapeutic strategies to mitigate silica-induced lung diseases.

## Data Availability

The original contributions presented in the study are included in the article/**Supplementary Material**. Further inquiries can be directed to the corresponding authors.
