# Correction: Single-cell and spatial transcriptomics reveal a stress-induced EMT-like epithelial subset driving immune activation in silica-injured lung

**DOI:** 10.3389/fimmu.2025.1671133

**Published:** 2025-08-20

**Authors:** Tao Wang, Jianfeng Hao, Bing Li, Ahjol Hyraht, Jialing Wang, Henglei Xia, Qingbin Wu, Wei Gao, Congxia Chen, Chuanqing Yu, Xiuqun Gong, Ting Li, Mei Zhang, Yinghai Xie, Xinrong Tao

**Affiliations:** ^1^ The First Hospital of Anhui University of Science and Technology (Huainan First People’s Hospital), Huainan, Anhui, China; ^2^ School of Public Health, Anhui University of Science and Technology, Hefei, Anhui, China; ^3^ School of Medicine, Anhui University of Science and Technology, Huainan, Anhui, China; ^4^ State Key Laboratory of Primate Biomedical Research, Institute of Primate Translational Medicine, Kunming University of Science and Technology, Kunming, China; ^5^ Kunshan Integrated Traditional Chinese and Western Medicine Hospital, Kunshan, Jiangsu, China

**Keywords:** silicosis, alveolar epithelial cells, single-cell RNA sequencing, spatial transcriptomics, epithelial-immune crosstalk

An incorrect grant number was provided for funder “Anhui Provincial Special Program for Clinical and Translational Medical Research”.The correct number is “No. 202304295107020033”.

The original version of this article has been updated.

