# Supplementary material for: Single-cell and spatial transcriptomics reveal a stress-induced EMT-like epithelial subset driving immune activation in silica-injured lung
Source: Front Immunol. 2025 Jun 6;16:1609616. doi: 10.3389/fimmu.2025.1609616 (PMC12179088; doi:10.3389/fimmu.2025.1609616)
Supplement: Supplementary file 1 [file DataSheet1.docx]

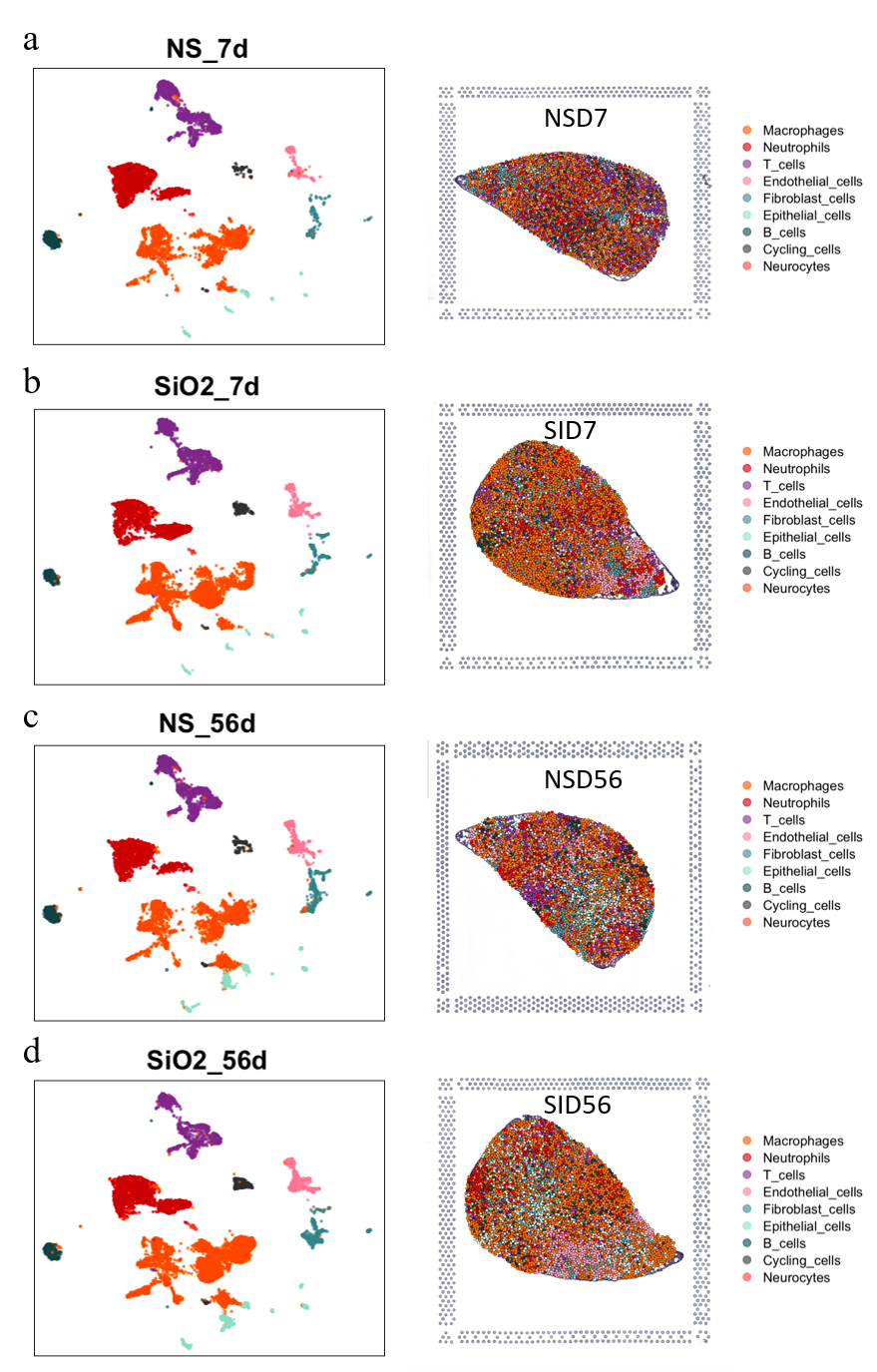


Supplementary Figure 1. UMAP Plots (left) and Spatial Distribution of Epithelial Cells (right) for Each Group at 7 and 56 Days Post-treatment: NS_7d (d), SiO₂_7d (e), NS_56d (f), and SiO₂_56d (g).


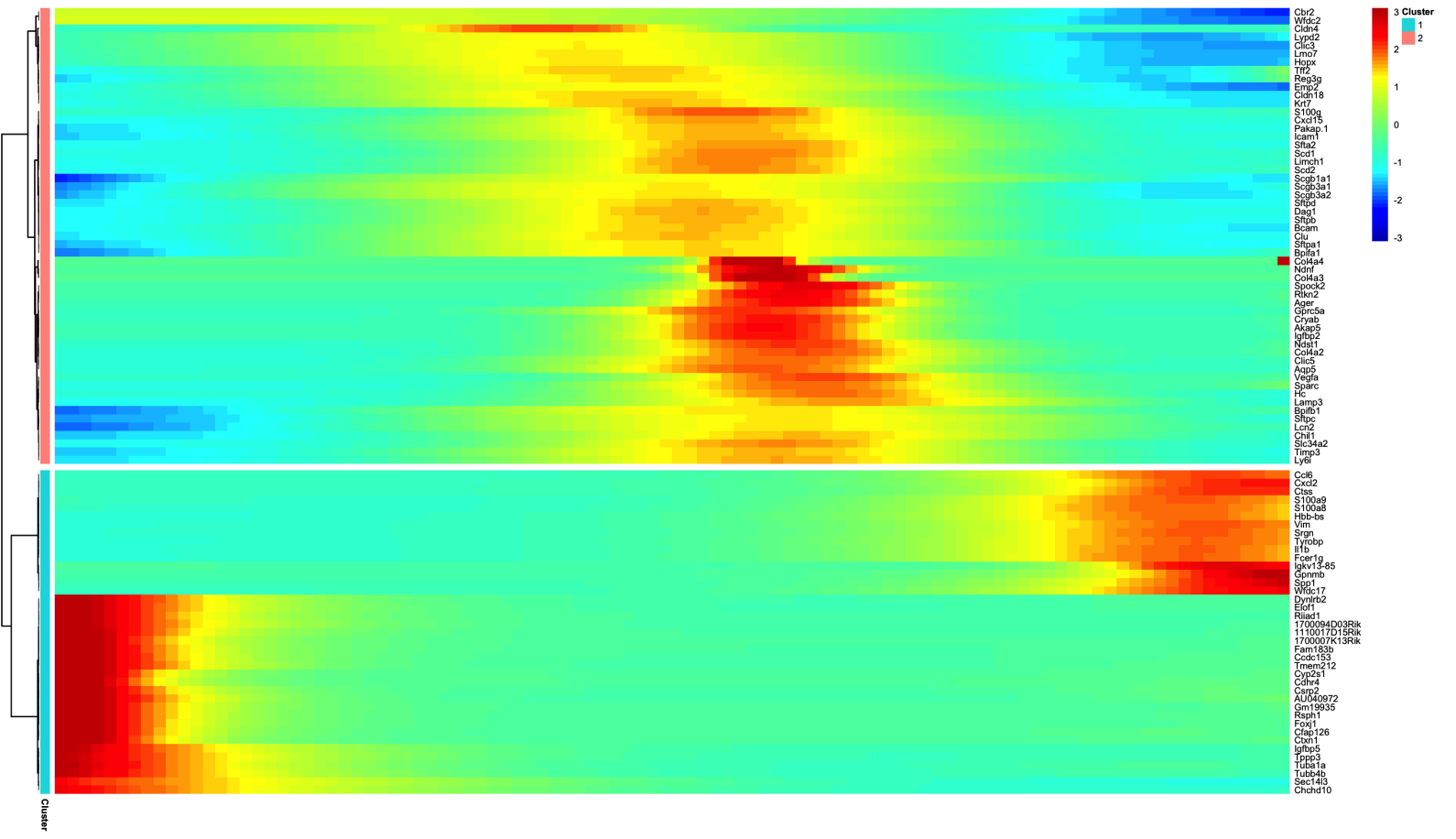


Supplementary Figure 2. Pseudotime heatmap of Differentially Expressed Genes across Epithelial Subpopulations (C0-C5) over The Course of Pseudotime Trajectory.


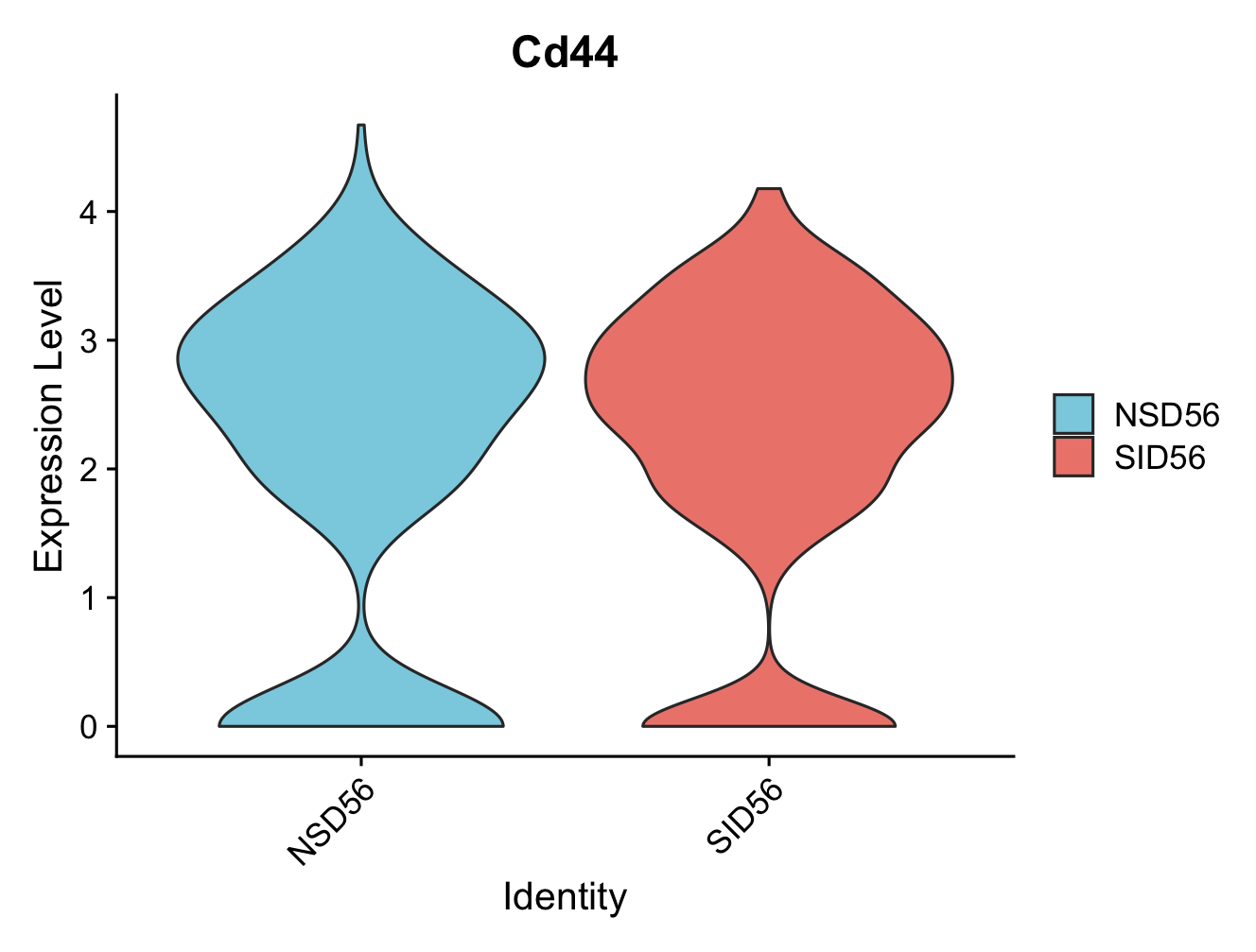

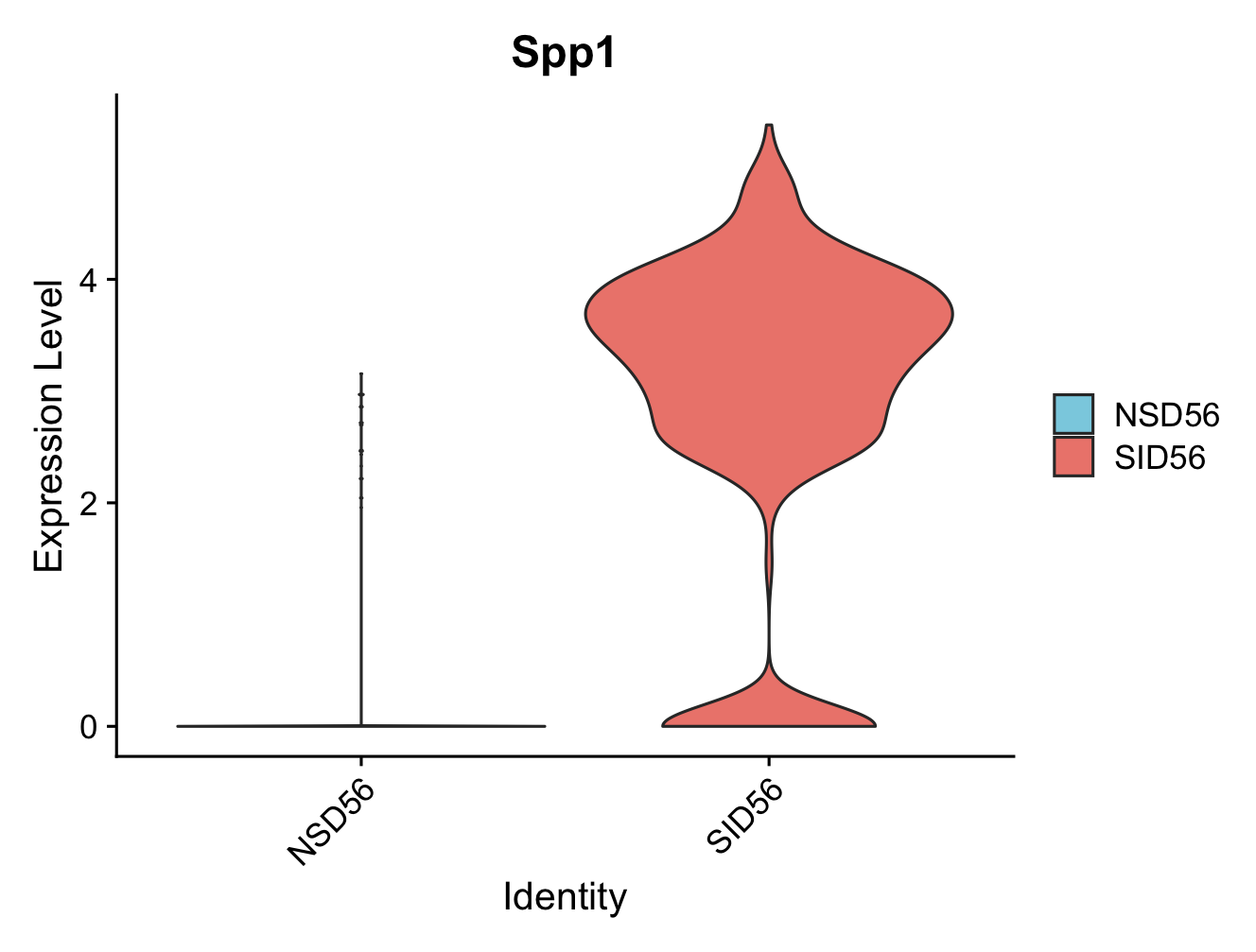


a

b

c


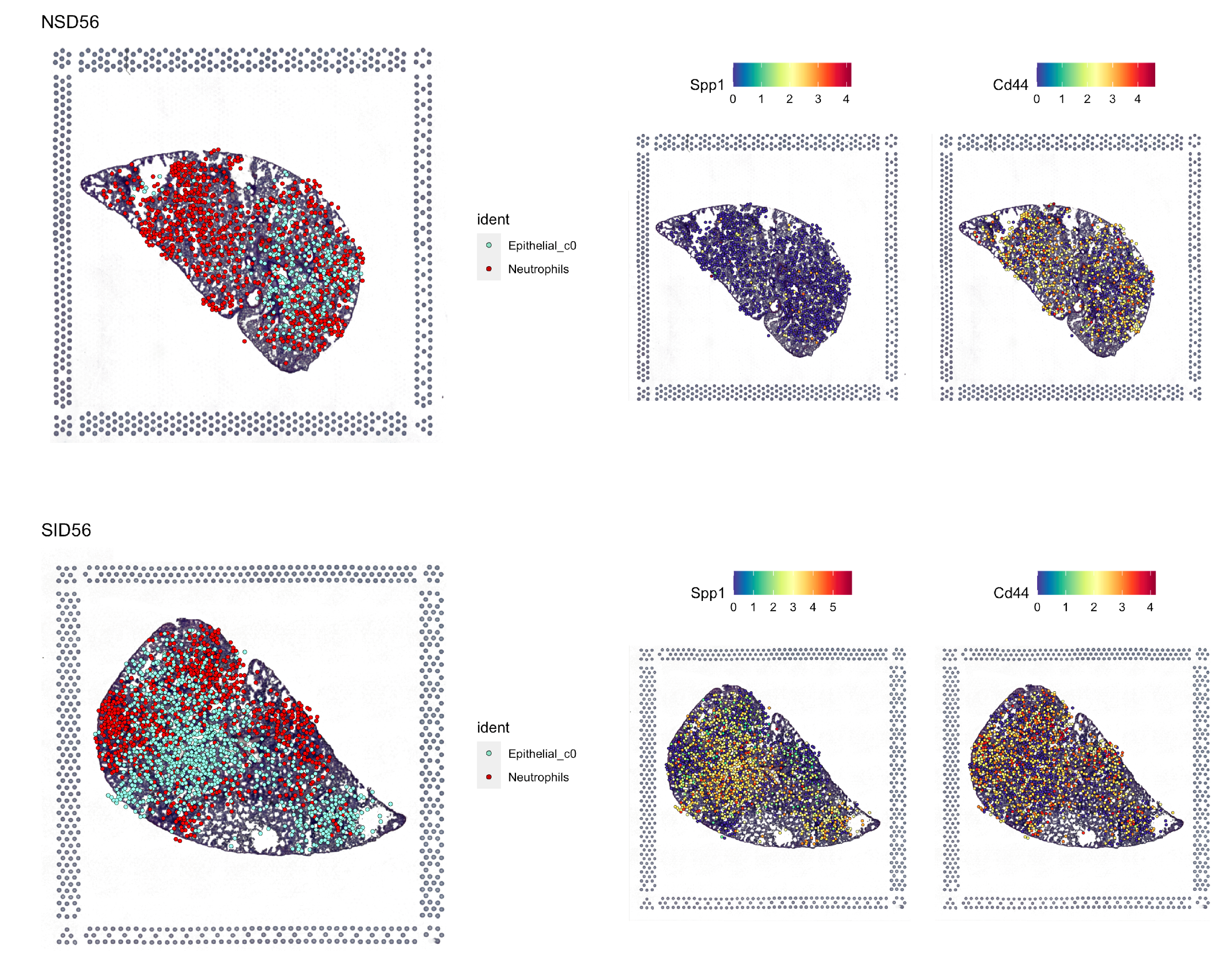


Supplementary Figure 3. Spatial Expression of Neutrophil Markers ( *Spp1*, and *Cd44* ) in NS_56d and Sio2_56d Tissues.


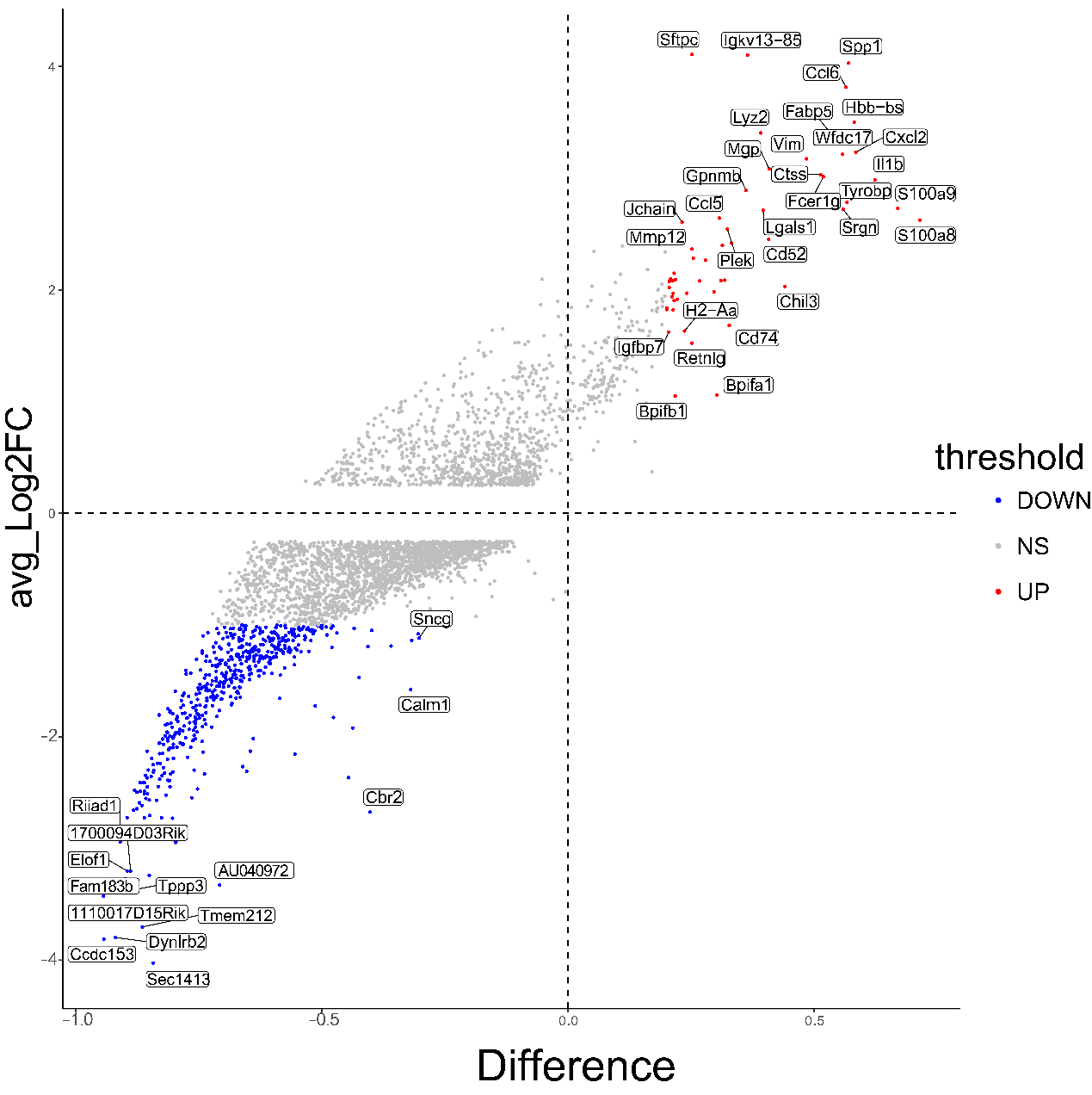


Supplementary Figure 4. Differential Expression Analysis between C0 and C1 Clusters (Mean-difference Plot). Red and Blue Dots Represent Significantly Upregulated and Downregulated Genes in C0 Relative to C1.
